# Identification of *NPR2* gene mutations affecting chondrocyte differentiation in short stature through JAK2-STAT5

**DOI:** 10.1186/s13023-025-03936-5

**Published:** 2025-08-01

**Authors:** Shuoshuo Wei, Mingming He, Chuanpeng Zhang, Yanying Li, Mei Zhang, Xinguo Hou, Bo Ban, Qianqian Zhao

**Affiliations:** 1 Department of Endocrinology, Genetics and Metabolism, Affiliated Hospital of Jining Medical University, Jining Medical University, 89 Guhuai Road, Jining, 272029 Shandong People’s Republic of China; 2 Medical Research Center, Affiliated Hospital of Jining Medical University, Jining, 272029 Shandong People’s Republic of China; 3 Chinese Research Center for Behavior Medicine in Growth and Development, Jining, 272029 Shandong People’s Republic of China; 4 Department of Endocrinology, Qilu Hospital of Shandong University, Jinan, 250012 Shandong People’s Republic of China

**Keywords:** Short stature, NPR2, Recombinant human growth hormone, JAK2-STAT5, Chondrocyte differentiation

## Abstract

**Background:**

Natriuretic peptide receptor 2 (*NPR2*) is a crucial regulator of endochondral bone growth. However, patients carrying heterozygous *NPR2* gene mutations exhibit a wide range of clinical phenotypes, and evidence regarding treatment efficacy is limited, with the pathogenic mechanisms not yet fully understood. Therefore, the aim of this study is to analyze and identify the clinical phenotypes, treatment outcomes, and pathogenic molecular mechanisms associated with *NPR2* gene mutations.

**Methods:**

Through exome sequencing, we sequenced *NPR2* in three Chinese Han patients with short stature and validated the results in their families. Clinical characteristics, treatment follow-up analysis, protein 3D structure prediction, in vitro functional experiments, and transcriptome sequencing were used to examine the protein changes caused by the variants, their pathogenicity, and the underlying molecular mechanisms of the disease.

**Results:**

All three patients with *NPR2* (p.R318W, p.I908T, p.R976H) gene mutations exhibited non-specific skeletal dysplasia and short stature, with good efficacy of recombinant human growth hormone (rhGH) treatment. Compared to the wild type, the protein expression level of *NPR2* mutants was significantly reduced (*P* < 0.001), and CNP-induced cyclic guanosine monophosphate (cGMP) production was significantly decreased (*P* < 0.0001). Transcriptome sequencing analysis revealed that *Csf2* is a key differentially expressed gene between *NPR2* mutant and wild type, and also an upstream regulator of the JAK2-STAT5 pathway. Further validation via qRT-PCR and Western blot showed that *NPR2* gene mutations significantly reduced *Csf2* gene mRNA expression (*P* < 0.05), and protein expression of JAK2, p-JAK2, and p-STAT5 was significantly decreased (*P* < 0.05). Further analysis revealed that *NPR2* gene mutations significantly affected the expression of chondrocyte differentiation markers *Sox9*, *Col2A1*, and *BMP4* (*P* < 0.001).

**Conclusion:**

Our study provides new insights into the loss of function of *NPR2*. *NPR2* gene mutations may influence the expression and phosphorylation levels of proteins in the JAK2-STAT5 signaling pathway by downregulating *Csf2*, thereby affecting chondrocyte differentiation and ultimately leading to short stature.

**Supplementary Information:**

The online version contains supplementary material available at 10.1186/s13023-025-03936-5.

## Background

The linear growth of stature is intricately dependent on the regulation of the growth plate, a cartilage zone bridging the epiphysis and metaphysis, serving as the primary differentiation site for longitudinal bone growth. The growth plate is composed of various cell types, with chondrocytes being paramount in this process [1]. Chondrocytes facilitate the repair and renewal of cartilage tissue through cellular proliferation and differentiation. Based on chondrocyte morphology, the bone growth plate is categorized into the resting zone, proliferative zone, prehypertrophic zone, hypertrophic zone, and terminal zone [2]. Notably, c-type natriuretic peptide (*CNP*) and natriuretic peptide receptor 2 (*NPR2*), expressed primarily in proliferating chondrocytes and pre-hypertrophic chondrocytes of the growth plate and is a key regulatory factors in endochondral ossification [3, 4]. The CNP-NPR2 signaling axis stimulates chondrocyte and cartilage matrix production, thereby promoting growth plate development [5, 6].

NPR2, a transmembrane protein, comprises an extracellular ligand-binding domain, a transmembrane domain, an intracellular homodimeric kinase domain, and a carboxy-terminal guanylate cyclase domain [3]. CNP, via autocrine or paracrine mechanisms, binds to NPR2, enhancing cartilage matrix synthesis and stimulating chondrocyte proliferation and hypertrophy. This interaction activates intracellular guanylate cyclase activity, leading to increased cyclic guanosine monophosphate (cGMP) production, which subsequently exerts its biological functions to promote bone elongation [7]. Pathogenic variations in the *NPR2* gene can give rise to a wide spectrum of phenotypic outcomes, ranging from severe limb girdle shortening caused by homozygous or compound heterozygous mutations to isolated short stature associated with heterozygous mutations [8]. Among children initially diagnosed with idiopathic short stature (ISS), *NPR2* heterozygous mutations have been identified in 2–6% of cases, while their prevalence is even higher in patients with non-syndromic familial short stature (FSS) [9, 10].

Previous studies have shown that mutations in the *NPR2* gene affect chondrocyte proliferation, differentiation and cartilage matrix synthesis by affecting the normal function of the CNP/cGMP signaling pathway, which ultimately leads to short stature [11]. Moreover, emerging research suggests that *NPR2* can impact chondrocyte function via endoplasmic reticulum stress, contributing to short stature as well [12]. However, the precise pathogenic mechanisms and functional consequences of altered *NPR2* genes in chondrocytes remain incompletely understood.

This study identified three mutations in the *NPR2* gene among three families with short stature. We conducted a thorough investigation into the clinical characteristics, laboratory test results, genetic information, treatment approaches, and follow-up data of three patients with short stature harboring *NPR2* gene mutations. Additionally, a series of functional analyses and transcriptome sequencing were performed to explore the potential pathogenic mechanisms underlying short stature caused by mutations in the *NPR2* gene.

## Material and methods

### Subjects

The patients were from the Department of Endocrinology and Genetic Metabolic Diseases at Jining Medical University Affiliated Hospital. Whole exome sequencing (WES) was conducted on three independent Chinese families with short stature, where the diagnostic criteria included: height standard deviation scores (SDS) ≤ − 2, and absence of chromosomal abnormalities. This study was approved by the Ethics Committee of Jining Medical University Affiliated Hospital. Written informed consent was obtained from all participants or their legal guardians.

### Clinical evaluations

Assessments included medical history, anthropometric measurements, laboratory tests, and radiographic examinations for all patients. Weight and height were accurately measured to calculate body mass index (BMI). Height SDS was calculated based on standard Chinese pediatric data [13]. Peak growth hormone (GH) was determined by levodopa and insulin hypoglycemic provocation test. Serum GH levels were quantified using chemiluminescence (Beckman Coulter, USA; sensitivity: 0.010 µg/L). Insulin-like growth factor 1 (IGF-1) levels were measured by chemiluminescent immunometric assay (Siemens, Germany; CV: internal 3.0%, external 6.2%) and normalized to SDS based on data from healthy peers [14]. Bone age was assessed using the Greulich and Pyle method on X-rays of the non-dominant hand [15]. During recombinant human growth hormone (rhGH) therapy, patients were followed up every 3 months. Two of the patients were followed up until adulthood to assess final adult height.

### Genetic examination

Genomic DNA was obtained from the proband and their parents using standard procedures from peripheral blood leukocytes. As previously described, WES was performed [16]. The *NPR2* gene was described according to the NCBI entry NG_009249 (NM_003995.4), and the sanger sequencing was performed to validate the candidate variant identifed.

### Construction of *NPR2* gene expression plasmid

The wild-type and mutant *NPR2* overexpression lentiviruses, as well as the empty vector (EV) lentivirus, were sourced from Shanghai Genechem Co., Ltd. The human WT and mutant *NPR2* gene sequences were inserted into the GV492-Flag expression vector. This vector carries a green fluorescent protein (GFP) gene for visualization and a puromycin resistance gene for selection. The constructs were transfected into HEK293T and ATDC5 packaging cells.

### Cell culture and transfection

HEK293T cells were cultured in high-glucose DMEM (Gibco, USA) with 10% fetal bovine serum (ExCell, China) at 37 °C in a 5% CO2 humidified atmosphere. ATDC5 cells were maintained in high-glucose DMEM/F12 (Gibco, USA) with 10% fetal bovine serum (ExCell, China) and 100 U/mL penicillin–streptomycin (Gibco, USA). ATDC5 cells were passaged every 2 days and induced to undergo chondrogenic differentiation in insulin-transferrin-selenium (ITS) (Sigma, USA) containing medium.

For the lentiviral vector expressing *NPR2*, cells were seeded in 6-well plates and transfected when they reached 60–80% confluence. The lentiviral vectors expressing *NPR2* were transfected into HEK293T or ATDC5 cells at MOI = 5 and MOI = 30, respectively. After 48 h of transfection, cell samples were collected and analyzed by Western blot using Flag antibody. Stable cells were selected with puromycin at a final concentration of 5 μg/mL (MCE, USA), and stable cell lines were established after 48 h. GFP expression was observed under a fluorescence microscope. Cells with the empty lentiviral vector were defined as vector controls.

### Quantitative real-time polymerase chain reaction (qRT-PCR)

Total RNA was isolated using TRIzol (Ambion, USA), followed by reverse transcription into cDNA with HiScript III RT SuperMix for qPCR (Vazyme, China). Quantitative PCR (qPCR) was prepared using Taq Pro Universal SYBR qPCR Master Mix (Vazyme, China) and detected on an ABI QuantStudio5 detection system. To verify the effect of *NPR2* mutants on chondrocyte differentiation, we examined the expression of chondrocyte markers, including *Col2A*, *BMP4*, and *Sox9*. The relative expression levels of mRNA were determined using the 2^−∆∆CT^ method. All experiments were performed in triplicate with *GAPDH* serving as the internal control. All primers for PCR amplifcation were designed using NCBI Primer-BLAST and purchased from Shanghai Sangon Biotechnology. The primer sequences are shown in Supplementary Table 1. Primers were designed using Primer Premier 6 and synthesized by Sangon Biotech Company (Shanghai, China).

### Western blotting

Cells were lysed with RIPA Lysis Buffer (P0013B, Beyotime, China) and protease inhibitors (P1045, Beyotime, China). Protein concentrations were determined using a BCA Protein Assay Kit (P0010, Beyotime, China). Equal amounts of protein (40 μg) were loaded onto a 7.5% sodium dodecyl sulfate–polyacrylamide gel electrophoresis (SDS-PAGE) gel and subsequently transferred onto a polyvinylidene fluoride (PVDF) membrane (Millipore, USA). The membrane was blocked with TBS-T containing 5% skimmed milk for 1 h at room temperature, followed by overnight incubation at 4°C with primary antibodies against Flag (1:1000, Sigma), actin (1:7500, ProteinTech), and GAPDH (1:50,000, Cell Signaling Technology). After washing three times with TBS-T buffer, the membrane was incubated with secondary antibodies for 1 h at room temperature. The membrane was rinsed again and then incubated with the SuperFemto ECL Chemiluminescent Kit (Vazyme, China). Signals were visualized using a Tanon 5800 Imaging System (Shanghai, China). Protein band intensities were further quantitatively analyzed using Image J software (NIH, USA).

The Plasma Membrane Protein Isolation Kit (Beyotime, China) was used to obtain cell membrane and cytoplasmic proteins. The protein extracts underwent electrophoresis and subsequent blotting as previously described. Actin (1:7500, ProteinTech) served as an internal control for cytoplasmic proteins, while ATP1A1 (1:1000, Affinity) was used as an internal control for cell membrane proteins.

### Immunofluorescence imaging of intracellular localization

Stably transfected HEK293T cells were seeded onto six-well plates and fixed with 4% paraformaldehyde for 20 min. Subsequently, the cells were permeabilized with 0.5% Triton X-100 for 10 min, followed by blocking with 5% BSA (Solarbio, China) for 1 h. Primary antibody staining was performed using Anti-Flag antibody (1:100, Proteintech, China) at 37 °C for 2 h. CoraLite594-conjugated goat anti-mouse IgG (H + L) secondary antibody (Proteintech, China) was then applied at a 1:500 dilution and incubated at room temperature for 1 h. Finally, cells were counterstained with DAPI (Beyotime, China) to visualize the nuclei. Fluorescent images were captured simultaneously in the red, green, and blue channels using a confocal laser scanning microscope (LSM800, Carl Zeiss, Germany) equipped with a 20 × objective lens. The ratio of cytoplasmic to nuclear fluorescence intensity was determined using ZEN software (Zeiss, Germany).

### Enzyme-linked immunosorbent assay

Stably transfected HEK293T cells were seeded onto six-well plates and washed twice with PBS after 24 h. Subsequently, 100 nM CNP (CNP-22; CAYMAN, Ann Arbor, MI, USA) was dissolved in serum-free DMEM and added to the cells. After incubation at 37 °C for 30 min, 0.1 M HCl was added to stop the activity of endogenous phosphodiesterases, thereby stabilizing cGMP. Cell lysates were collected, and cGMP levels were detected using the cGMP Complete ELISA Kit (ENZO Life Sciences, Madison Avenue, NY, USA).

### High-throughput sequencing and identification of differentially expressed (DE) mRNA

To detect changes in the transcriptome profiles of mutant and wild-type *NPR2*, RNA was extracted from induced and differentiated mutant and wild-type ATDC5 cells using TRIzol reagent (Invitrogen, CA, USA). The integrity of the RNA was assessed using a Bioanalyzer 2100 (Agilent, CA, USA). mRNA was isolated using Dynabeads Oligo (dT) (Thermo Fisher, USA) and fragmented using a magnesium-based fragmentation kit (NEB, cat.e6150s, USA) at 94 °C for 5–7 min. The fragmented mRNA fragments were then reverse transcribed using SuperScript™ II Reverse Transcriptase (Invitrogen, cat.1896649, USA) to generate a cDNA library. The library was subjected to paired-end sequencing using an Illumina Novaseq™ 6000, following standard operating procedures.

### Statistical analysis

The values are presented as mean ± standard deviation. All data were analyzed using GraphPad Prism 8.0.2 software. One-way ANOVA was used for comparisons among more than two groups. Statistical significance was considered when the *p* value was less than 0.05. The 3D structures of *NPR2* wild type and mutants were predicted using AlphaFold and Pymol software. Sequencing data were aligned to the genome using HISAT2. Differential gene analysis among samples was performed using the R package edgeR, with a fold change > 2 or < 0.5 and a *p* value < 0.05. Finally, gene ontology (GO) and Kyoto Encyclopedia of Genes and Genomes (KEGG) enrichment analyses were conducted using DAVID software (https://david.ncifcrf.gov/).

## Results

### Clinical and genetic characteristics

**Fig. 1:**
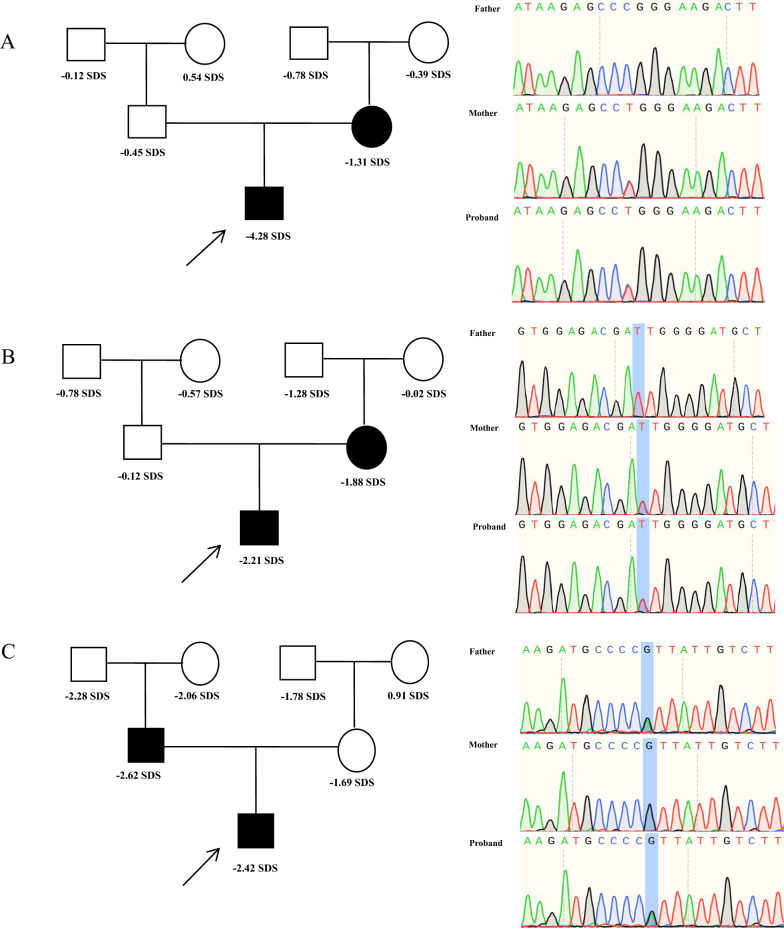
Family map of patients with *NPR2* gene variants and validation by Sanger generation sequencing. **A**: patient 1 (p.R318W); **B**: patient 2 (p.I908T); **C**: patient 3 (p.R976H)

All three patients carrying heterozygous *NPR2* gene variants were boys, aged between 9.9 and 14.4 years, with height SDS ranging from − 2.21 to − 4.28. They had normal body proportions, with two patients presented with short fingers, and one patient showed elbow valgus (Table 1). Exome sequencing and Sanger sequencing revealed that the *NPR2* gene variants in all three patients were inherited from their parents, with two variants inherited from the mothers and one from the father (Fig. 1).

**Table 1:** Clinical characteristics of patients with *NPR2* gene mutation

Variable	Patient 1	Patient 2	Patient 3
Genetic information	NPR2 c.952C>T, p.R318W	NPR2 c.2723T>C, p.I908T	NPR2 c.2927G>A, p.R976H
Inherited	Mother	Mother	Father
Gender	Boy	Boy	Boy
Age (years)	14.4	9.9	12.4
Bone age (years)	14.0	7.0	10.0
Height (cm)	139.1	126.5	137.0
Height SDS	− 4.28	− 2.21	− 2.42
Sitting height/height	0.54	0.51	0.53
Height/arm span	1.01	1.04	1.07
Body weight (kg)	35	21.5	30
BMI (kg/m^2^)	18.09	13.44	15.98
Birth length (cm)	50	50	50
Birth weight (kg)	3.3	2.5	3.8
Father's height (cm)	170	172	157
Mother's height (cm)	153	150	151
MPH (cm)	168	167.5	160.5
IGF-1 (ng/mL)	240.7	214.0	186.0
IGF-1 SDS	− 0.74	− 0.24	− 1.20
Peak GH (ng/mL)	7.21	5.51	14.02
Clinical phenotype	Elbow extensor	Short fingers	Short fingers

SDS, standard deviation score; BMI, body mass index; IGF-1, insulin-like growth factor 1; IGF-1 SDS, insulin-like growth factor 1 standard deviation score; NPR2, natriuretic peptide receptor 2; GH, growth hormone

**Fig. 2:**
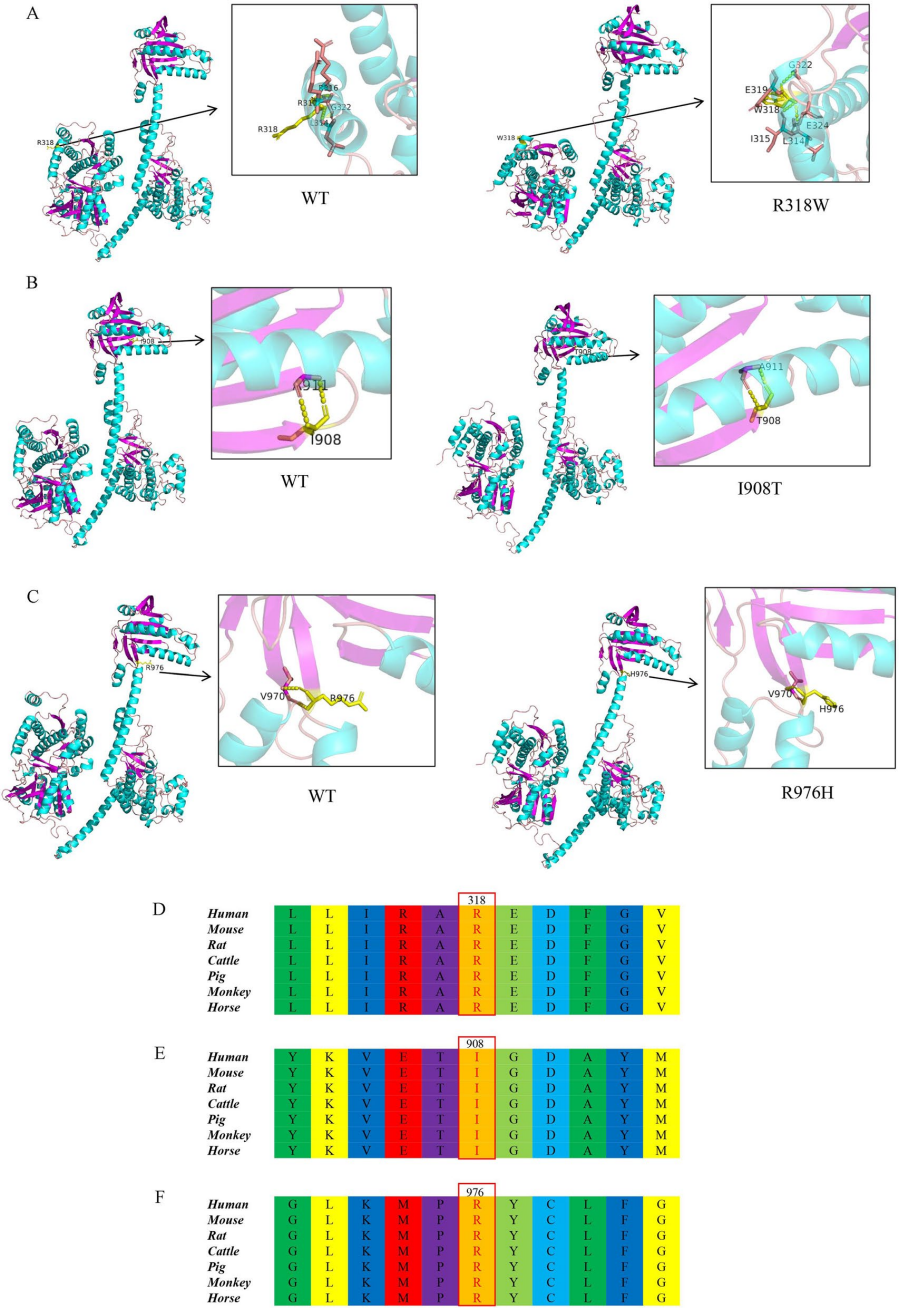
Three-dimensional structure prediction and conservativeness analysis of mutant proteins in the *NPR2* gene. Three-dimensional structure prediction: **A**: p.R318W; **B**: p.I908T; **C**: p.R976H. Conservativeness analysis: **D**: p.R318W; **E**: p.I908T; **F**: p.R976H

In addition, three-dimensional structure prediction of the proteins of wild-type and mutant types of *NPR2* using Alphafold and Pymol software revealed that the structural and spatial positions, such as helix and curl, were altered in the mutant type of *NPR2* (Fig. 2A–C), and the conserved analyses showed that the three variants were highly conserved among different species (Fig. 2D–F).

**Fig. 3:**
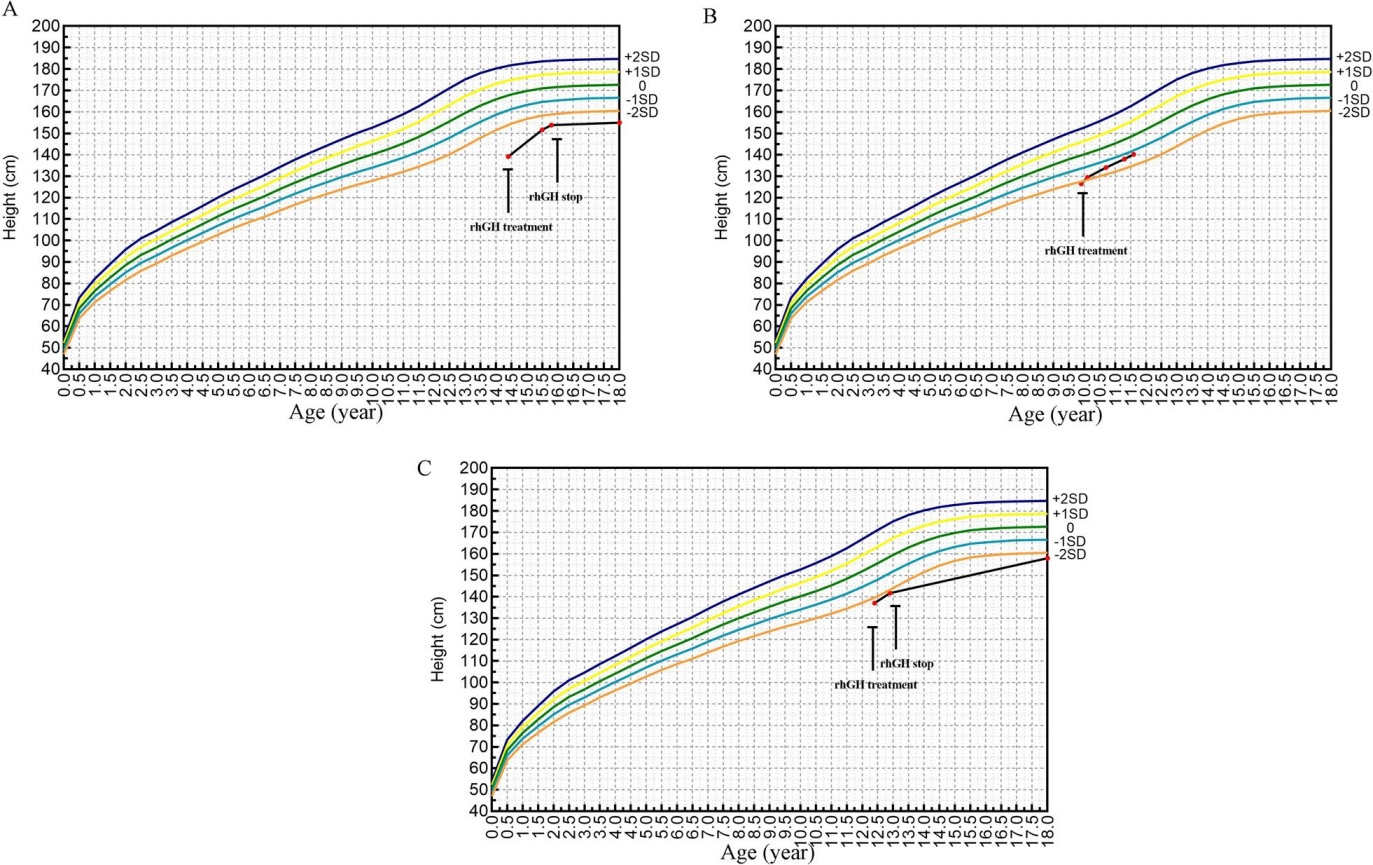
Improvement in height with rhGH treatment in patients with *NPR2* gene variants. **A**: p.R318W; **B**: p.I908T; **C**: p.R976H

All three patients underwent rhGH treatment for durations ranging from 0.5 to 2 years, resulting in increases in height SDS by 0.14 to 1.41, respectively. Among them, two patients were followed up to their final adult height, with height SDS of − 2.95 and − 2.45, respectively (Table 2). The growth curves of the patients are depicted in Fig. 3.

**Table 2:** Follow-up of rhGH in children with *NPR2* gene variants

Treatment	Patient 1	Patient 2	Patient 3
Age at start of treatment (years)	14.4	9.9	12.4
Duration of treatment (years)	1.5	2	0.5
Age after treatment (years)	15.9	11.8	12.9
Height at start of treatment (cm)	139.1	126.5	137
Treatment initiation HtSDS	− 4.28	− 2.21	− 2.42
Height after treatment (cm)	153.8	140.2	141.7
HtSDS after treatment	− 2.87	− 1.17	− 2.28
ΔHeight (cm)	14.7	13.7	4.7
ΔHtSDS	1.41	1.04	0.14
Follow-up adult height (cm)	155	–	158
Adult HtSDS	− 2.95	–	− 2.45

HtSDS, height standard deviation score; rhGH, recombinant human growth hormone; NPR2, natriuretic peptide receptor 2

### Genetic variation affects transcription, translation and intracellular localization of *NPR2*

**Fig. 4:**
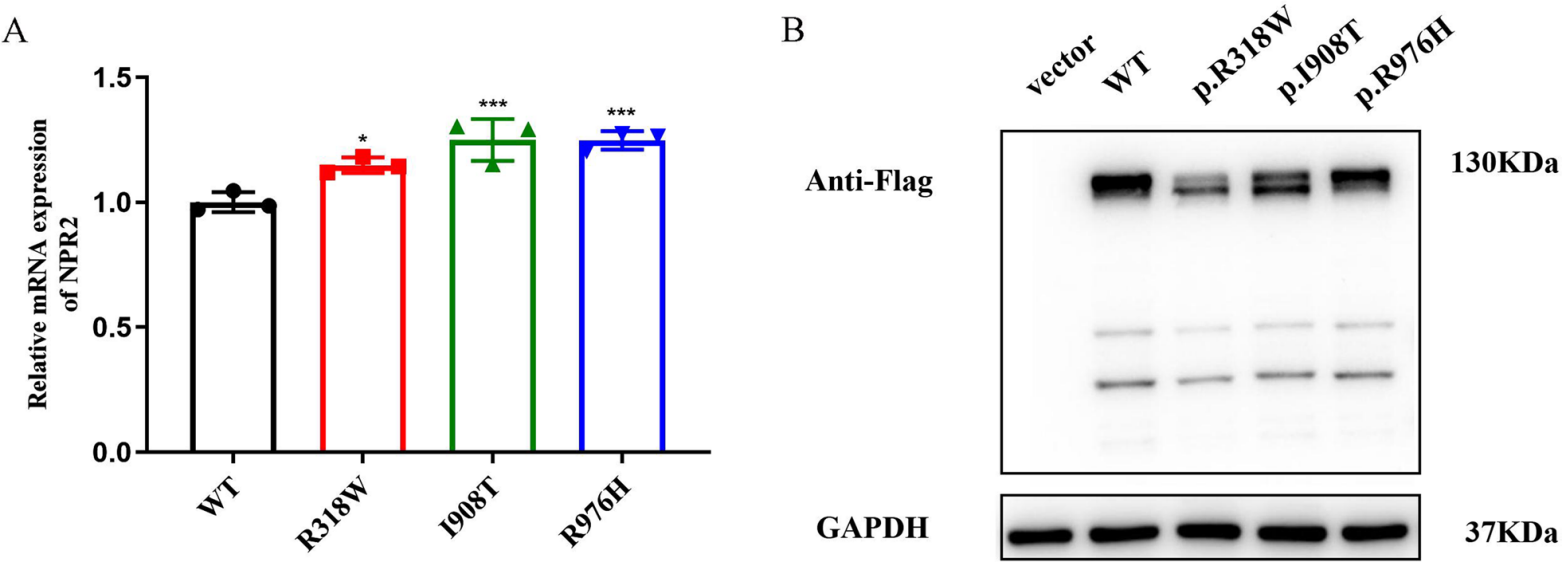
Transcription and protein expression of mutant and wild-type *NPR2* genes in HEK293T cells. **A**: Relative quantitative analysis of mRNA levels of WT and mutant *NPR2* by qRT-PCR; **B**: Expression of WT and mutant NPR2 protein by Western blot

**Fig. 5:**
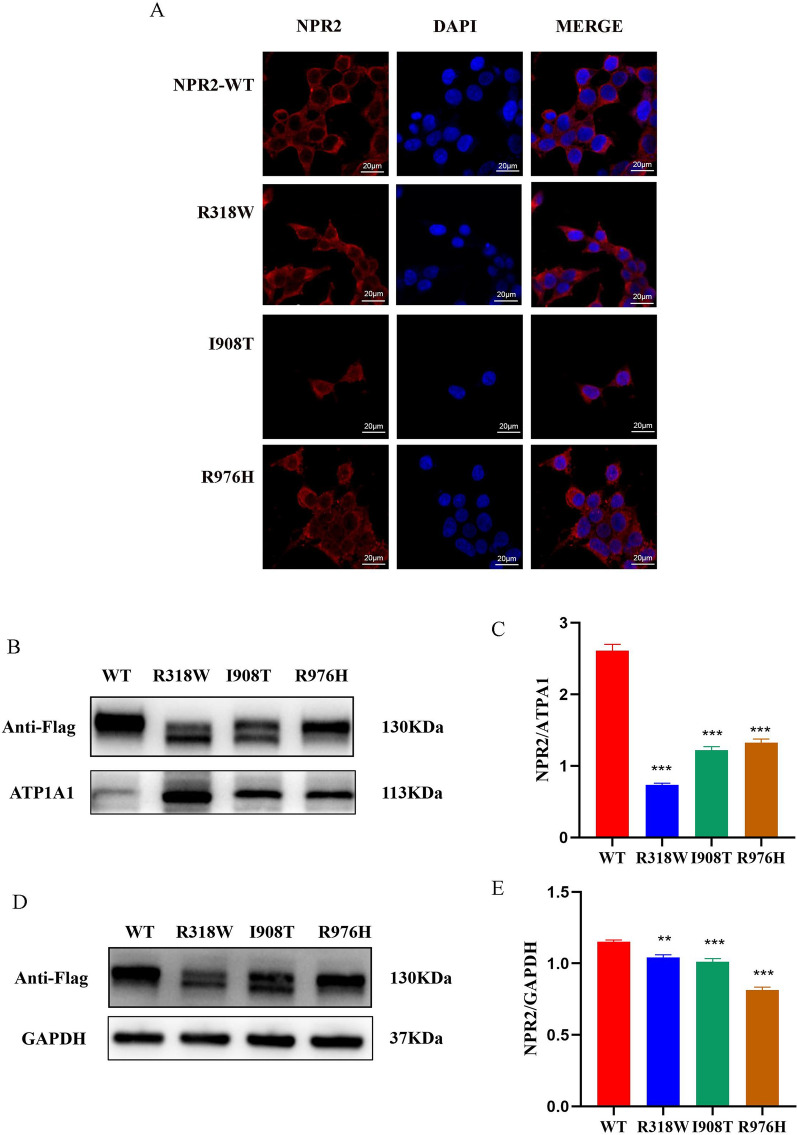
Subcellular localization of NPR2 proteins in HEK293T cells. **A**: Confocal microscopy images showed the localization of both wild-type and mutant NPR2 proteins; **B**: Cell membrane expression of wild-type and mutant NPR2 proteins detected by Western blot. **C**: Analysis of NPR2 protein expression in cell membrane by Western blot using Image J; **D**: Cytoplasmic expression of wild-type and mutant NPR2 proteins detected by Western blot; **E**: Analysis of NPR2 protein expression in cytoplasmic by Western blot using Image J

The qRT-PCR results showed that the mRNA levels of the *NPR2* mutant were significantly higher compared to the wild-type *NPR2* (*p* < 0.0001) (Fig. 4A). The Western blotting analysis indicated that although the molecular weights of the NPR2 missense mutations (p.R318W, p.I908T, p.R976H) were similar to that of the wild-type NPR2, the protein expression levels were significantly reduced (Fig. 4B). Confocal fluorescence microscopy images revealed that the protein expressed by the wild-type *NPR2* gene was evenly distributed in the cytoplasm and on the cell membrane. However, the proteins expressed by the *NPR2* gene mutations R318W, I908T, and R976H accumulated in the cytoplasm and formed aggregates (Fig. 5A). Western blot analysis showed that the expression levels of the R318W, I908T, and R976H mutant proteins of the *NPR2* gene on the cell membrane were significantly reduced compared to the wild-type (Fig. 5B, C). Although there was a slight decrease in the expression levels of the mutant NPR2 proteins compared to the wild-type in the cytoplasm (Fig. 5D, E), the significant reduction in overall protein expression indicated that these mutations affect the transport of NPR2 protein to the cell membrane. This further demonstrates the abnormal subcellular localization of the mutant proteins.

### Mutations in the *NPR2* gene affect cGMP levels

**Fig. 6:**
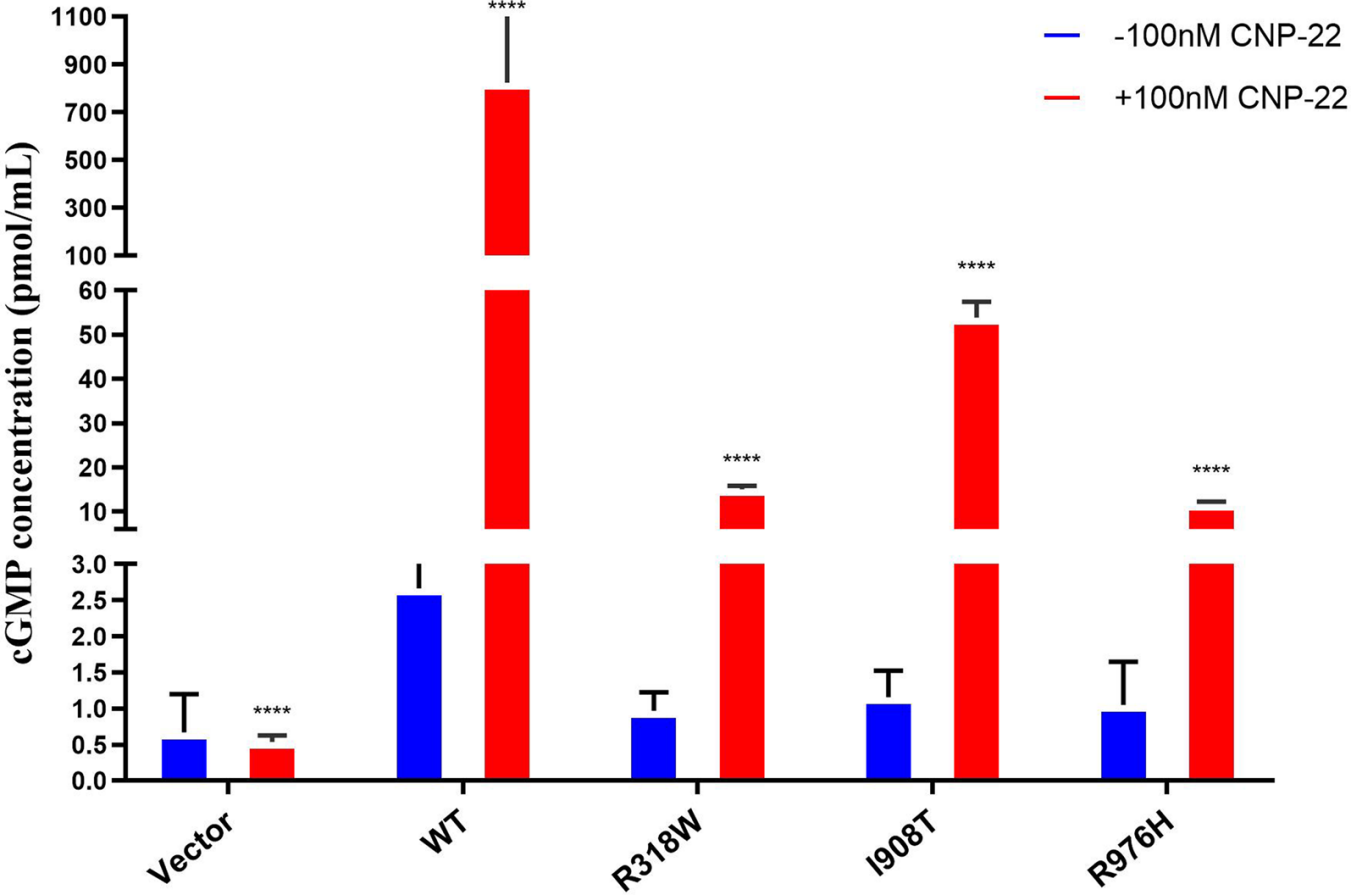
Impaired cyclic guanosine monophosphate (cGMP) production by NPR2 mutations. ****P < 0.0001

Figure 6 evaluates the signaling capability mediated by *NPR2* gene mutations in response to CNP. The results indicate that, compared to the functionally normal wild-type *NPR2*, the mutant *NPR2* significantly reduced cGMP production upon CNP induction. This finding suggests that mutations in the *NPR2* gene may impair the signaling efficiency after CNP binds to its receptor, thus affecting the regulation of intracellular cGMP levels. Furthermore, even in the absence of CNP induction, the mutant *NPR2* exhibited lower baseline levels of cGMP, further implying that the mutant *NPR2* may negatively impact intracellular cGMP homeostasis. Due to the limitation of samples, we further examined the serum of patients carrying *NPR2* gene mutations in 2 cases, while selecting 3 normal children of the same age and gender as controls. Compared to normal children, the serum cGMP concentration in *NPR2* gene mutation (p.I908T, p.R976H) patients was significantly decreased (Supplementary Fig. 1).

### *NPR2* influences chondrocyte differentiation through the JAK2-STAT5 signaling pathway

**Fig. 7:**
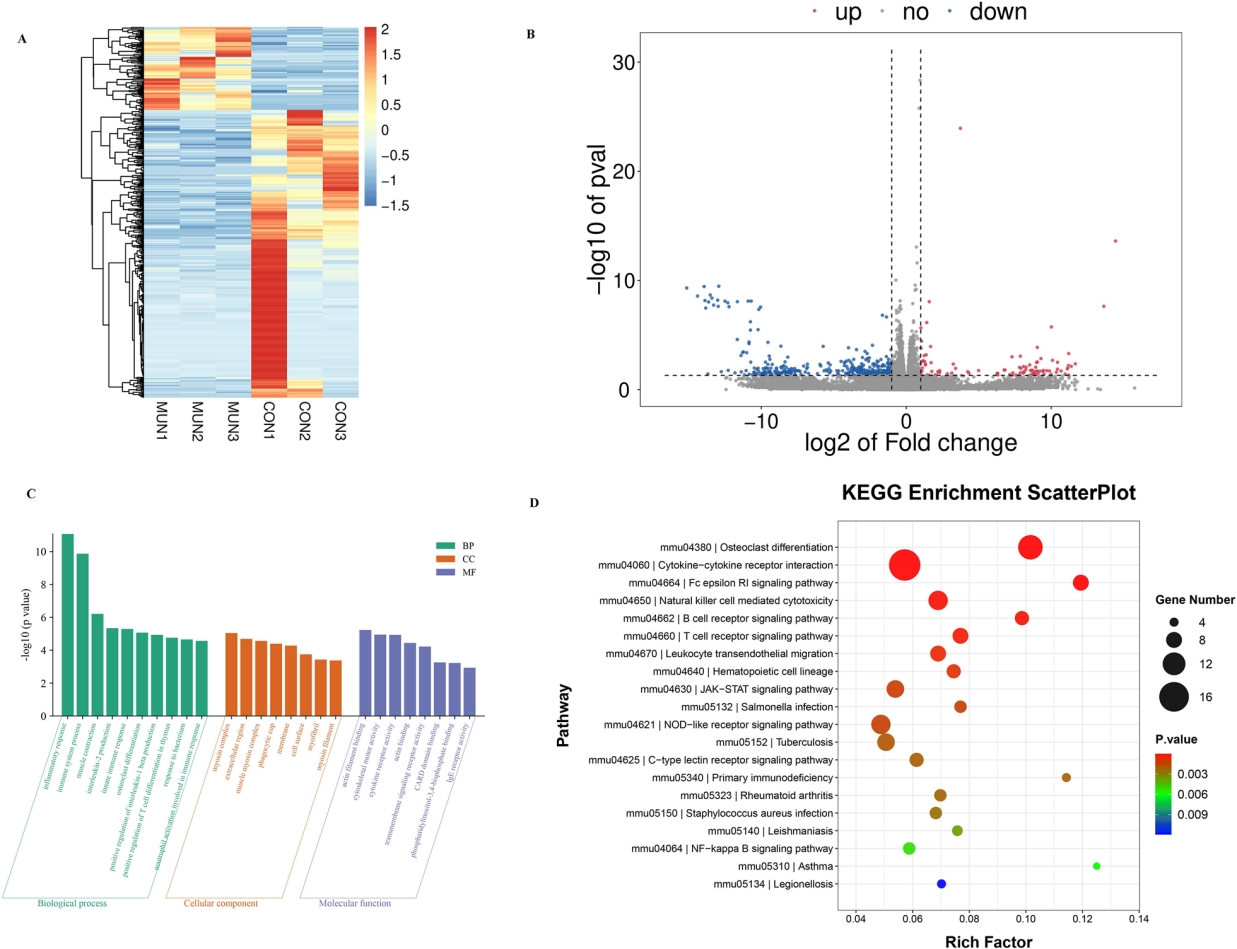
Differential expression profile and functional analysis of mRNA in chondrogenic differentiation of ATDC5 cells with *NPR2* gene mutation. **A**: Cluster diagram of differential genes; **B**: Volcano plot of differential genes; **C**: GO analysis of differential genes involved in biological processes; **D**: KEGG enrichment of differentially expressed genes involved in signaling pathways

The mRNA expression profiles of *NPR2* mutants and wild types exhibit significant differences, as illustrated in Fig. 7A. A total of 447 genes with significantly altered expression were identified, comprising 100 upregulated genes and 347 downregulated genes, as depicted in Fig. 7B. The GO enrichment analysis revealed that Biological Process (BP) encompassed inflammatory responses and immune processes, with predominant enrichment in Cellular Component (CC) such as the actin-myosin complex and extracellular domains. Molecular Function (MF) was predominantly associated with actin filament binding and cytoskeletal motility activity (Fig. 7C). Additionally, to investigate the functionality and interactions of differentially expressed genes, KEGG pathway enrichment analysis was conducted. This analysis indicated that the JAK-STAT signaling pathway is closely related to growth (Fig. 7D).

**Fig. 8:**
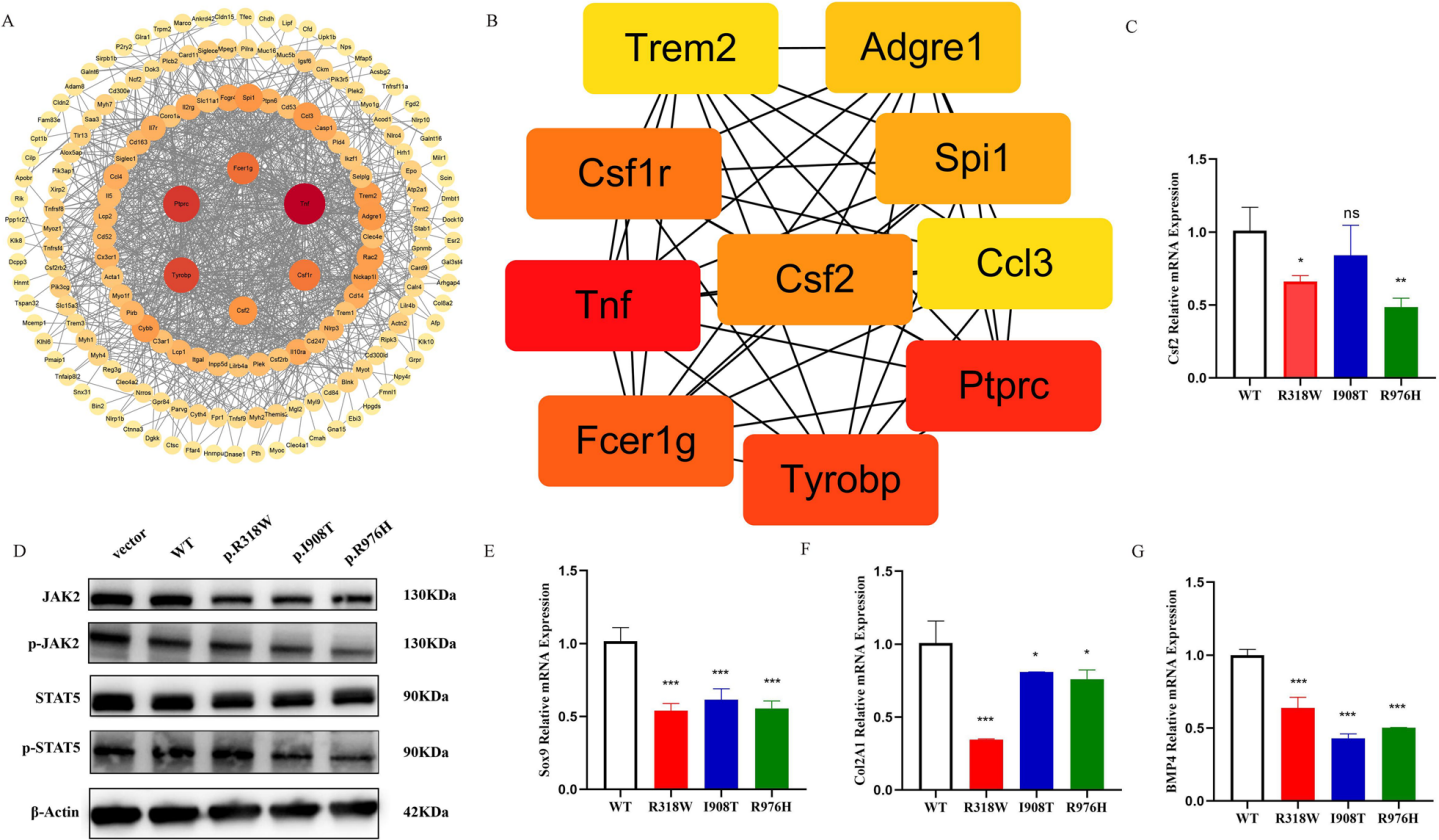
Effects of *NPR2* gene variation on *csf2* and JAK2-STAT5 signaling pathways and chondrogenic differentiation markers. **A**: Analysis of protein–protein interactions using the string database and cytoscape software; **B**: Identification of the top 10 hub proteins through the cytoHubba plugin; **C**: mRNA expression of *csf2* gene; **D**: Western blot analysis of JAK2-STAT5 total protein and phosphorylated protein expression in *NPR2* gene mutation; **E**: mRNA expression of *Sox9* gene; **F**: mRNA expression of *Col2A1* gene; **G**: mRNA expression of *BMP4* gene. ns = no statistically significant difference, *P < 0.05; **P < 0.01; ***P < 0.001

By employing string to predict protein–protein interactions, a protein–protein interaction network was constructed (Fig. 8A), with Csf2 identified as a key protein (Fig. 8B). Additionally, *Csf2* serves as an upstream regulatory gene in the JAK2-STAT5 signaling pathway, and the activation of *Csf2* can trigger a series of downstream cellular responses and transcriptional programs by activating the JAK2-STAT5 signaling pathway. The results of qRT-PCR analysis revealed that compared to the wild-type *NPR2* gene, the mutation of the *NPR2* gene significantly reduced the mRNA expression of the *Csf2* gene (*P* < 0.05), as shown in Fig. 8C.

Further Western blot analysis indicated that *NPR2* gene mutation led to a significant reduction in the protein expression levels of JAK2 and p-JAK2, while the I908T and R976H mutations of the *NPR2* gene caused a significant decrease in the protein expression of p-STAT5 (Fig. 8D). This suggests that *NPR2* gene mutations may influence the phosphorylation levels of JAK2 and STAT5 by downregulating the *Csf2* gene. Additionally, the examination of chondrocyte differentiation markers *Sox9*, *Col2A1*, and *BMP4* revealed that *NPR2* gene mutations significantly affected the expression of these chondrocyte differentiation markers (Fig. 8E–G). These results further support the notion that *NPR2* gene mutations may impact the differentiation process of chondrocytes by affecting the expression and phosphorylation levels of key factors such as Csf2, JAK2, and STAT5, thereby leading to related features such as short stature.

## Discussion

The C-type natriuretic peptide receptor encoded by the *NPR2* gene is a paracrine regulator of the growth plate and plays a crucial role in human skeletal development. Heterozygous variants of the *NPR2* gene can lead to short stature and may result in varying degrees of bone development abnormalities. This study found that the protein expression levels associated with *NPR2* gene mutations were significantly decreased, and CNP-induced cGMP production was markedly reduced. Further analysis indicated that *NPR2* gene mutations may affect the protein expression and phosphorylation levels of the JAK2-STAT5 signaling pathway by downregulating *Csf2*, thereby impacting chondrocyte differentiation and ultimately leading to the occurrence of short stature. These findings provide important insights into the mechanisms by which *NPR2* gene mutations influence short stature.

This study reports two cases of familial short stature associated with heterozygous mutations in the *NPR2* gene, which are linked to short stature with nonspecific clinical manifestations [10]. Patient 1 presented with short stature and elbow extensor, while Patients 2 and 3 presented with short stature and short fingers. The penetrance of *NPR2* heterozygous mutations is high, but there is significant variability in clinical manifestations among patients, with the majority exhibiting short stature without skeletal dysplasia. Previous study reports that patients carrying *NPR2* gene mutations exhibit a wide range of clinical phenotypes, including varying degrees of high-arched palate, mid-limb shortening, cubitus valgus, delayed bone age, shortened fourth metacarpal, cone-shaped epiphyses, and short fifth fingers [17]. Hisado-Oliva et al. screened 697 individuals with disproportionate short stature and their family members, and first reported that heterozygous mutations in NPPC lead to short stature in affected patients and their relatives, with affected family members also exhibiting small hands and mild facial abnormalities [18]. Previous studies indicate that heterozygous mutations in the *NPR2* gene may result in patients with short stature presenting with different characteristics, considering the severity of short stature, body proportions, and various nonspecific skeletal abnormalities [19].

Recent studies have indicated that there are significant differences in the efficacy of rhGH therapy among short patients with *NPR2* gene mutations [20, 21]. Vasque et al. found that rhGH could not significantly increase the height of larger age children with *NPR2* heterozygous mutations [21]. Additionally, other study has shown that rhGH treatment can significantly increase the height of patients with *NPR2* heterozygous mutations [9]. All three patients in this study received rhGH treatment. Significant improvement in height was observed with regular rhGH therapy, whereas poor improvement was seen with discontinued or irregular rhGH treatment. A recent study investigated the clinical characteristics and rhGH treatment efficacy of three patients with *NPR2* gene mutations. The study found that after 2 years of rhGH therapy, the average improvement in HtSDS was 1.59 ± 0.1 SDS, indicating a good response to rhGH treatment among the three patients with *NPR2* gene mutations [22]. As individuals with *NPR2* heterozygous gene mutations age, their potential for height gradually diminishes, leading to an increased severity of short stature [23]. To prevent final adult short stature, early application of rhGH therapy is crucial. However, the efficacy of rhGH treatment in patients with *NPR2* heterozygous variations still requires long-term follow-up studies in a larger population.

Functional studies have shown that compared to the wild-type, cells transfected with the *NPR2* gene mutation exhibit a significant decrease in cGMP levels both before and after CNP stimulation, indicating that the *NPR2* gene mutation has a dominant negative effect. This is consistent with previous studies [12]. The *NPR2* protein consists of four structural domains, including the extracellular ligand-binding domain, transmembrane domain, kinase homology domain, and guanylyl cyclase domain. Previous studies have confirmed that functional mutations in the kinase homology domain of NPR-B increase cGMP activity compared to the wild-type protein [24]. Most *NPR2* gene variants associated with ISS lead to abnormal protein transport to the plasma membrane, reduced affinity of CNP for ligands, or inhibition of *NPR2* activity [25]. Immunofluorescence and Western blot analyses in this study show that *NPR2* gene mutant proteins fail to be successfully transported to the cell membrane, resulting in decreased expression of cell membrane *NPR2* protein, and accumulation of the protein on one side of the cytoplasm during protein expression, further confirming that *NPR2* gene mutations lead to abnormal protein transport to the plasma membrane and reduced affinity of CNP for ligands. The cellular effects of CNP action are mediated through the NPR-B receptor, stimulating the production of intracellular cGMP, which in turn activates protein kinases G (PKG). Activation of CNP leads to PKG activation, initiating the MAPK signaling pathway and ultimately promoting chondrocyte proliferation and differentiation [26].

This study further conducted transcriptome sequencing on successfully induced stable transfected *NPR2* wild-type and mutant chondrocytes. Through KEGG pathway enrichment analysis, it was found that mutations in the *NPR2* gene might affect the activation of the JAK-STAT signaling pathway. The JAK-STAT pathway is an important signaling pathway that plays a crucial role in various biological processes, including cell growth, differentiation, and survival [27]. Specifically, during skeletal growth, the normal activation of the JAK2-STAT5 signaling pathway is essential for maintaining the differentiation of cells in the growth plate [28]. A recent study has shown that abnormalities in the JAK2-STAT5B signaling pathway can inhibit the proliferation of chondrocytes [29]. The study results suggest that the JAK2-STAT5 signaling pathway may play a role in the skeletal regulatory functions of *NPR2*. *NPR2* plays an important role in skeletal development by regulating chondrocyte proliferation and differentiation, as well as bone growth [26]. This study indicates that *NPR2* gene mutations may mediate the regulation of chondrocyte differentiation through the JAK2-STAT5 signaling pathway, thereby affecting skeletal development. *NPR2* activates the corresponding signaling pathways by producing cGMP [4], and cGMP, as a second messenger, may be involved in activating JAK2, subsequently triggering a series of reactions leading to the phosphorylation of STAT5. The phosphorylated STAT5 translocates to the nucleus, where it activates the transcription of target genes, thereby influencing skeletal development.

## Conclusions

In conclusion, this study describes the clinical characteristics and rhGH treatment responses of three patients with *NPR2* gene mutations, and reveals that *NPR2* gene mutations can significantly affect protein expression, cellular localization, and cGMP levels. By successfully inducing chondrocyte differentiation for signal molecule pathway studies, the results show that *NPR2* gene mutations can influence chondrocyte differentiation by downregulating *Csf2*, thereby affecting the phosphorylation levels of JAK2 and STAT5, which in turn leads to short stature.

## Supplementary Information

Additional file 1.

## Data Availability

The datasets used and/or analysed during the current study are available from the corresponding author on reasonable request.

## Abbreviations

NPR2: Natriuretic peptide receptor 2
CNP: C-type natriuretic peptide
cGMP: Cyclic guanosine monophosphate
GH: Growth hormone
IGF-1: Insulin-like growth factor 1
WES: Whole exome sequencing
rhGH: Recombinant human growth hormone
GFP: Green fluorescent protein
qRT-PCR: Quantitative real-time polymerase chain reaction
GO: Gene ontology
BP: Biological process
KEGG: Kyoto Encyclopedia of Genes and Genomes
Csf2: Colony-stimulating factor 2
PKG: Protein kinases G
