# Supplementary material for: Identification of NPR2 gene mutations affecting chondrocyte differentiation in short stature through JAK2-STAT5
Source: Orphanet J Rare Dis. 2025 Aug 1;20:392. doi: 10.1186/s13023-025-03936-5 (PMC12315304; doi:10.1186/s13023-025-03936-5)
Supplement: Supplementary file 1 — Additional file 1. [file 13023_2025_3936_MOESM1_ESM.doc]

**Supplementary table 1 Sequences of PCR primers**

| Target gene | Forward primer (5′–3′) | Reverse primer (5′–3′) |
| --- | --- | --- |
| *Gapdh* (Human) | ACAACTTTGGTATCGTGGAAGG | GCCATCACGCCACAGTTTC |
| *Gapdh* (Mouse) | GGAGCCAAAAGGGTCATCATCT | GAGGAGCCATCCACAGTCTTCT |
| *NPR2* (Human) | ACCTCATCGCTGGCTGCTTCTA | CCCGTCCACCAAATCTGCTTCT |
| *Sox9* (Human) | ATGACCGACGAGCAGGAGAAGG | TCGCTTGACGTGTGGCTTGTTC |
| *Col2a1* (Human) | GTGGAGCAGCAAGAGCAAGGA | TCAGTGGACAGTAGACGGAGGA |
| *BMP4* (Human) | CACAGCGGTCCAGGAAGAAGAA | GCACAATGGCATGGTTGGTTGA |
| *Csf2* (Mouse) | TGTGGTCTACAGCCTCTCAGCA | GGCATGTCATCCAGGAGGTTCA |


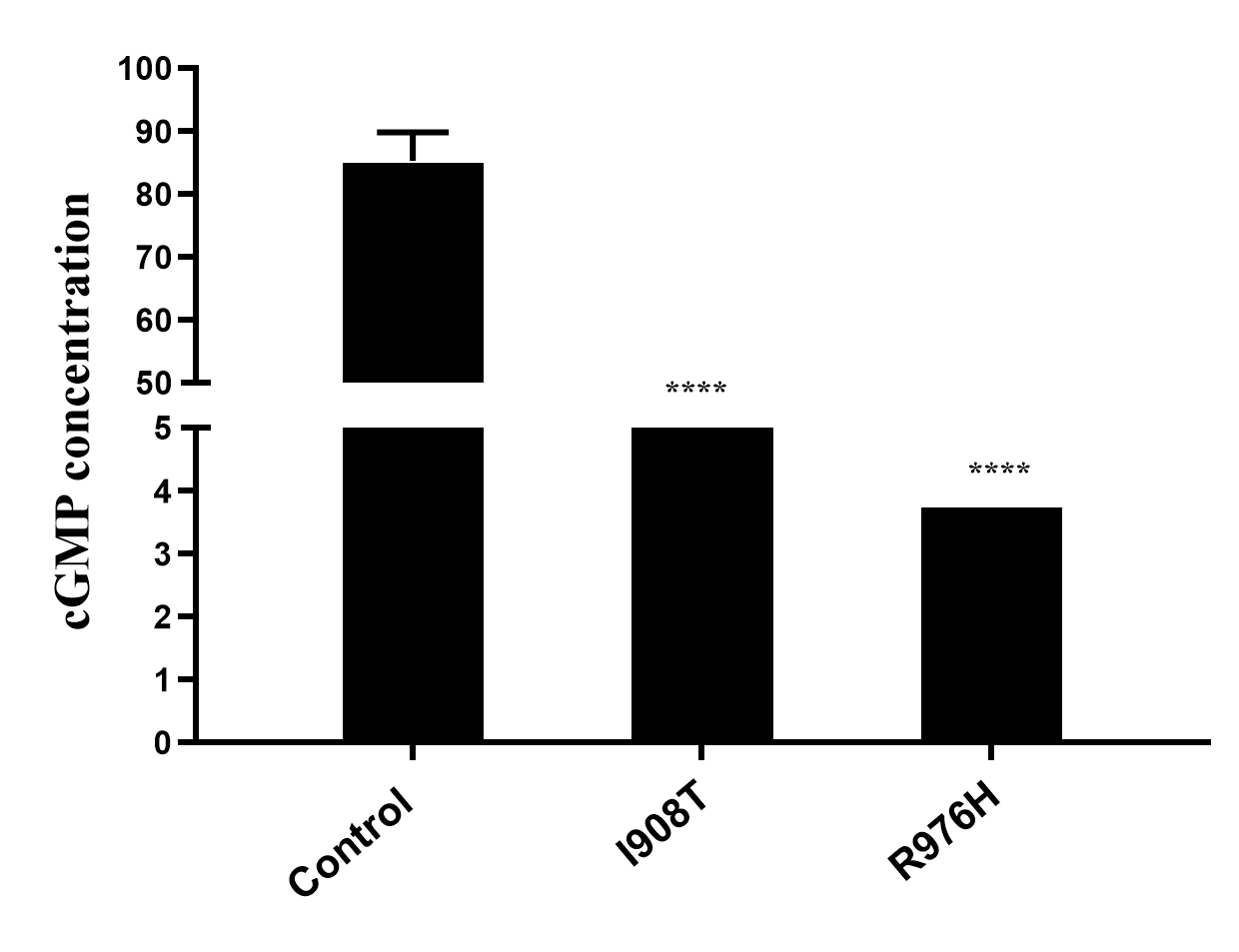


Supplementary Figure 1. The mutation of *NPR2* gene in patients resulted in the dysgenesis of human serum cyclic guanosine monophosphate (cGMP). ****P<0.0001
